# Rational design of antibodies and development of a novel method for (1–3)-β-D glucan detection as an alternative to Limulus amebocyte lysate assay

**DOI:** 10.3389/fcimb.2024.1322264

**Published:** 2024-01-24

**Authors:** Chunlong Liu, Lin Zhang, Jiaxing Zhang, Mengfan Wang, Shengping You, Rongxin Su, Wei Qi

**Affiliations:** ^1^ Chemical Engineering Research Center, School of Chemical Engineering and Technology, Tianjin University, Tianjin, China; ^2^ R&D Department, Dynamiker Biotechnology (Tianjin) Co., Ltd, Tianjin, China; ^3^ Tianjin Key Laboratory of Membrane Science and Desalination Technology, Tianjin University, Tianjin, China; ^4^ State Key Laboratory of Chemical Engineering, Tianjin University, Tianjin, China; ^5^ Collaborative Innovation Center of Chemical Science and Engineering (Tianjin), Tianjin, China

**Keywords:** invasive fungal infections, (1-3)-β-D glucan, antibody rational design, rapid detection, limulus, chemiluminescence

## Abstract

With advances in medicine, increasing medical interventions have increased the risk of invasive fungal disease development. (1-3)-β-D glucan (BDG) is a common fungal biomarker in serological tests. However, the scarcity of Limulus resources for BDG detection poses a challenge. This study addresses the need for an alternative to Limulus amebocyte lysate by using BDG mutant antibody for chemiluminescence detection. The wild-type BDG antibody was obtained by immunizing rabbits. An optimal V52^H^I/N34^L^ Y mutant antibody, which has increased 3.7-fold of the testing efficiency compared to the wild-type antibody, was first achieved by mutating “hot-spot” residues that contribute to strong non-covalent bonds, as determined by alanine scanning and molecular dynamics simulation. The mutant was then applied to develop the magnetic particle chemiluminescence method. 574 clinical samples were tested using the developed method, with a cutoff value of 95 pg/mL set by Limulus amebocyte lysate. The receiver operating characteristic curve demonstrated an area under the curve of 0.905 (95% CI: 0.880–0.929). Chemiluminescence detected an antigen concentration of 89.98 pg/mL, exhibiting a sensitivity of 83.33% and specificity of 89.76%. In conclusion, the results showed a good agreement with Limulus amebocyte lysate and demonstrated the feasibility of using BDG mutant antibodies for invasive fungal disease diagnosis. The new method based on chemiluminescence for detecting BDG could shorten the sample-to-result time to approximately 30 min, rescue Limulus from being endangered and is resource efficient in terms of equipment and the non-use of a skilled technician.

## 1 Introduction

The increasing use of invasive tests, antibiotics, immunosuppressants, glucocorticoids, and chemotherapeutic agents has led to a rise in fungal infections, which poses a serious threat to public health. Each year, fungal diseases cause over 1.6 million death and severely affect more than 1 billion people worldwide (Almeida et al., 2019; Fisher et al., 2022). Fungi are diverse and adaptable organisms. They usually exist on human mucosal surfaces, but they can invade tissues and cause invasive fungal disease (IFD) in immunocompromised individuals (Salazar et al., 2022). IFD has typical clinical symptoms and is difficult to diagnose, leading to delayed treatment and severe consequences (Terrero-Salcedo and Powers-Fletcher, 2020). However, traditional diagnostic methods such as culture and histopathology have limitations in sensitivity and speed, making rapid and accurate microbiological diagnosis crucial (Ricna et al., 2019; Lass-Flörl et al., 2021). Serological diagnosis has become a popular auxiliary clinical diagnostic method for IFD with the advantages of high sensitivity, strong specificity, and convenience (Hage et al., 2019; Azap, 2020).

(1–3)-β-D glucan (BDG) is one of the fungal biomarkers widely used in serological detection (Mercier et al., 2019; Finkelman, 2021). Fungal Glucan Detection (G test) was then developed for testing the concentration of BDG in serum, which could help to diagnose IFD infection (Yoshida, 2021). The G test for the measurement of BDG uses the coagulation cascade mediated by factor G in the Limulus amebocyte lysate test (Yoshida, 2021). Unfortunately, the raw materials of Limulus amebocyte lysate come from natural Limulus resources, which are widely killed for their medicinal and edible value. In addition, the artificial cultivation of limulus is difficult, and large-scale cultivation has never been achieved (Kwan et al., 2018). With the increasing scarcity of Limulus resources, it is necessary to find a way to replace Limulus amebocyte lysate.

Chemiluminescence immunoassay, which is based on a fast “mix-and-measure” protocol, provides a promising automatic alternative for testing analytical procedures (Calero et al., 2022). Although it highly relies on instrument, chemiluminescence immunoassay still has significant advantages over other non-isotopic ELISA such as the high sensitivity and a wide linear range. In addition, compared with the Limulus amebocyte lysate method, the chemiluminescence method is free of endotoxin contamination. Most importantly, it has a sustainable raw material source, which offers a long-term development for the testing method. Therefore, this study aimed to develop a magnetic particle-based chemiluminescence immunoassay for the measurement of fungal BDG. The process was as follows (1): The wild-type antibody (WT-BDG-Ab) was obtained by immunizing rabbits and the full sequence plasmid expression vector was constructed (2). Alanine scanning of the antibody CDR region revealed seven residues (Trp33^H^, Val52^H^, Asp54^H^, Phe58^H^, Tyr99^H^, Pro105^H^, and Tyr106^H^) in the heavy chain and five residues (Tyr31^L^ Asn33^L^, Asn34^L^, Gly52^L^, and Arg55^L^) in the light chain as critical residues for antigen–antibody binding (3). The optimal mutant antibody V52^H^I/N34^L^Y was obtained by combined mutation screening (4). The V52^H^I/N34^L^Y mutant was applied to the chemiluminescence platform, and the results of 574 clinical samples showed a good correlation with the Limulus amebocyte lysate. This study provides a reasonable basis for the modification of WT-BDG-Ab to improve its potency and an effective detection method for the early diagnosis of IFD, which broadens the technical field of Limulus amebocyte lysate.

## 2 Materials and methods

### 2.1 Immunization and specificity analysis

Owing to the poor water solubility of standard BDG, carboxymethylated BDG was chosen as the immunogen, which was purchased from Megazyme (carboxymethylated substitution degree of 20%) and purified. It was mixed with Freund’s adjuvant complete in equal volumes. After full emulsification, New Zealand large-eared rabbits were injected subcutaneously at multiple points, and the immunization dose was controlled at 0.1–0.2 mg/time per rabbit. Ear blood was taken 3 days before immunization and the serum was isolated for negative control. After the initial immunization, the rabbits were immunized every 2 weeks in the same way. A total of eight immunizations were performed and the spleen was taken after the last immunization.

Enzyme-linked immunosorbent assay (ELISA) was used to analyze the specificity of the BDG antibody (BDG-Ab). Enzyme plates were coated with 50 ng/well of BDG, candida mannan, trehalose galactomannan, or cryptococcal polysaccharide, and the directly blocked plates were used as the negative control. The wells were blocked and incubated at 37°C for 1 h, with 100 μL of the serum which 16-fold diluted by 0.01 M PBS. They were washed and placed in 100 μL of goat anti-rabbit-HRP antibody for 30 min at 37°C, then washed and developed with 100 μL of tetraethylbenzidine (TMB) substrate for 15 min.

### 2.2 Sequence comparison and model construction of BDG-Ab

BDG-Ab sequencing was commissioned from GenScript (**Supplementary Table 1**, GenBank OR729008-OR729009). The IgBLAST system was used to compare the antibody libraries (Sayers et al., 2021). The heavy chain (BDG-Ab-H) and light chain (BDG-Ab-L) were submitted to the Kabat database, respectively, and the framework regions (FRs) and complementarity-determining regions (CDRs) in the variable region of BDG-Ab were identified.

The three-dimensional structure of BDG-Ab was constructed based on homology modeling and the machine learning method by trRosetta and Uni-Fold software, respectively (Du et al., 2021; Su et al., 2021). Since antibodies consist of two heavy and two light chains, homology modeling can only build single chains and then match the constructed single chains together. The machine learning method directly predicts the atomic coordinates of the antibody using a combination of its amino acid sequence, multiple sequence alignment, and solved homologous structures (Li et al., 2022).

### 2.3 Molecular docking and molecular dynamics simulations

The BDG model built by AmberTools was assigned the Amber ff99SB force field (Tian et al., 2020) and docked to the CDR region of BDG-Ab by Autodock vina (Eberhardt et al., 2021). GROMACS 2019.5 software was used for molecular dynamics (MD) simulations (Abraham et al., 2015). The simulation process was as follows (1): The water molecules and ions were modeled and inserted into the box to make the system charge equilibrium (2). The 2,000 energy minimization steps and 40-ns production simulations were applied to achieve the minimum energy point (3). The simulations were carried out in an isothermal–isobaric ensemble with periodic boundary conditions. The simulation parameters were a 25-fs step length, a 1-atm pressure, and a 298 K temperature, with the coupling constants of 12.0 and 1.0 ps, respectively. The simulation results were drawn and analyzed using Gromacs utility and VMD software (Humphrey et al., 1996). The binding energy of antibody and BDG was calculated by gmx_mmpbsa.py python script (Valdés-Tresanco et al., 2021).

### 2.4 Detection of the relative binding capacity of BDG-Ab mutant

**Figure 1:**
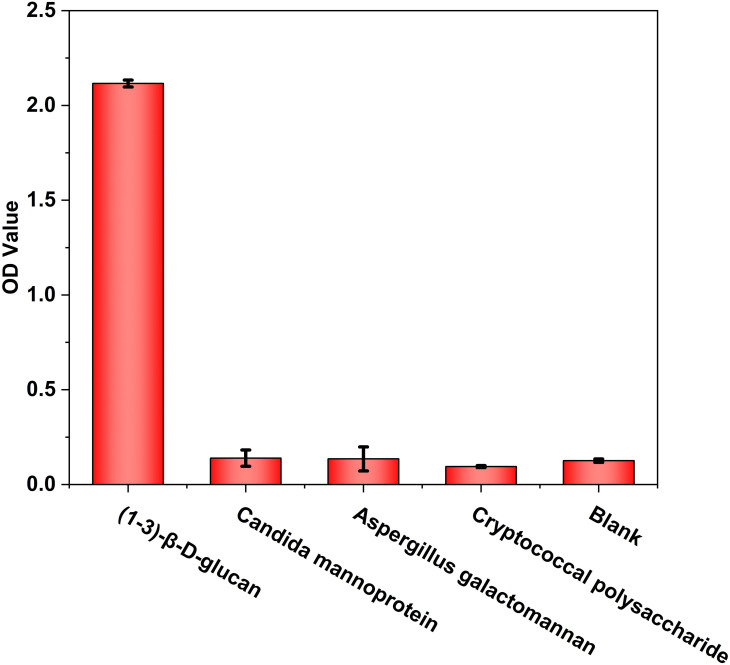
The specificity analysis of WT-BDG-Ab to different antigens using enzyme-linked immunosorbent assay (ELISA). In the blank control, the equal volume of endotoxin-free water was used instead of antigen.

**Figure 2:**
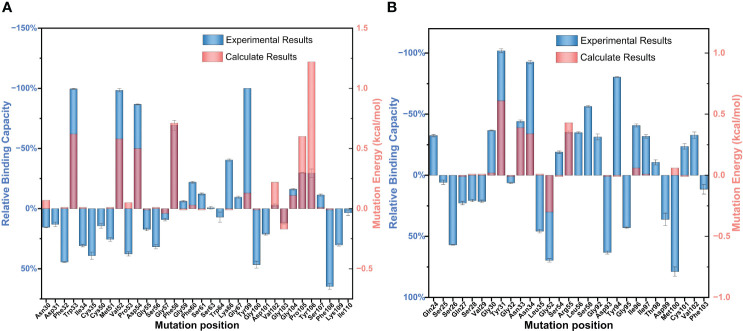
Partial results of the identification of BDG-Ab complementarity-determining region (CDR) residues involved in antigen binding via alanine scanning mutagenesis. The relative antigen binding of the BDG-Abs alanine substitution mutations on **(A)** heavy chain and **(B)** light chain was evaluated by indirect ELISA method and molecular dynamics simulation. Error bars represent standard deviations from the means for three independent experiments.

The sequences of BDG-Ab-H and BDG-Ab-L were ligated to the AbVec2.0 plasmid by Genewiz Biotech Co., Ltd (Suzhou, China) (**Supplementray Figures 1 and 2**). The non-alanine sequence in the CDR region of the BDG-Ab-H and BDG-Ab-L plasmids was sequentially point mutated to the alanine sequence. The mutated plasmids were provided by Genewiz Biotech Co., Ltd (Suzhou, China).

The mutated BDG-Ab-H/L plasmids paired with unmutated BDG-Ab-L/H plasmids were transfected into 293T cells. Cell supernatants were harvested after 48 h for two separate ELISA assays, in which enzyme plates were coated with BDG or mouse anti-rabbit lgG Fab antibody. The plates were added to the BDG-Ab separately, incubated at 37°C for 1 h, washed and placed in mouse anti-rabbit lgG Fc-HRP antibody for 30 min at 37°C, and then washed and developed with TMB substrate for 15 min at 37°C. The relative binding capacity (RBC) of BDG-Ab was calculated as:


$$RBC=\{(\frac{\frac{A}{B}}{\frac{{\text{A}}_{WT}}{B_{WT}}})-1\}\times 100\%$$

where *A* and *B* are the result of ELISA assay with BDG and mouse anti-rabbit lgG Fab antibody-coated enzyme plates, respectively, and WT is the wild-type BDG-Ab.

### 2.5 Potency testing of BDG-Ab mutants

BDG-Ab mutants were purified by the AmMagTM ProteinA Magnetic Beads (GenScript) kit after the plasmids were transfected into 293T cells. Antibody potency was verified by the indirect ELISA method. The ELISA plates were coated with 20 ng/well of BDG, and BDG-Ab mutants were diluted at concentrations of 1,000, 500, 250, 125, 62.5, 31.25, 15.625, 7.8125, and 3.90625 ng/mL, respectively. The plates were added to the dilutions separately, incubated at 37°C for 1 h, washed and placed in goat anti-rabbit-HRP antibody for 30 min at 37°C, and then washed and developed with TMB substrate for 15 min at 37°C. We defined the BDG-Ab potency as the reciprocal of the BDG-Ab concentration at OD = 0.

### 2.6 Fungi and bacteria recognizing ability comparison of wild-type and mutant BDG-Ab

Microplates were coated with filtered ultrasonic crushing solutions of different fungi and bacteria. Then, 100 ng/mL BDG-Ab mutants and wild-type BDG-Ab were added into the coated microplates to validate the recognition capability of different fungi and bacteria. In addition, the microplates coated with fungi- and bacteria-free ultrasonic crushing solution were used as negative control. *Aspergillus fumigatus, Aspergillus niger, Aspergillus terreus, Penicillium digitatum, Candida albicans, Pneumocystis jiroveci, Histoplasma capsulatum, Cyanobacteria marneffei, Cryptococcus neoformans, Saccharomyces cerevisiae, Trichophyton interdigitalis*, and *Cladosporium cladosporioides* were used in this research.

### 2.7 Comparison of the analytical capability of the Limulus amebocyte lysate method and the chemiluminescence method

According to EP 17 A2, the limit of blank (LoB), limit of detection (LoD) of the Limulus amebocyte lysate method, and the chemiluminescence method were compared. Precision comparison was conducted based on EP05 A3. The linearity evaluation was conducted referring to EP06 A. Long-term stability was tested according to EP25 A.

The chemiluminescence testing system was constructed using the following procedure. The BDG-Ab mutant diluent (1 mg/mL) was configured with 0.02 M PB (pH = 7.2) buffer, Sulfo-NHS-LC-Biotin (10 mg/mL) was added to 14.85 μL/mL, mixed for 2 h in the dark, and then dialyzed in 0.02 M PB (pH = 7.2) and labeled with biotin. Meanwhile, the BDG-Ab mutant diluent (1 mg/mL) was configured with 0.05 M CBS buffer, NSP-SA-NHS (2 mg/mL) was added to 45.5 μL/mL, mixed for 2 h in the dark, and then dialyzed in 0.02 M PB (pH 7.2) and labeled with acridine ester. A SMART 6500 automatic chemiluminescence detector was used for chemiluminescence detection. The clinical sample size was set to 100 μL and the amount of streptavidin-magnetic particles (0.4 mg/mL) was set to 40 μL. According to the requirement of antibody gradient concentration, the amount of two kinds of antibody master mixes that were added was adjusted. The fungal (1–3)-β-D-glucan assay kit [Dynamiker (Tianjin) Biotechnology Co. Ltd] was used for the Limulus amebocyte lysate method. The detailed operation method referred to protocol of the BDG testing kit. The LoB, LoD, linear range, and stability of the Limulus amebocyte lysate method and chemiluminescence method were compared. The sample volume for both BDG detention methods is 20 μL.

### 2.8 Clinical sample testing

A total of 574 serum samples were collected from Shandong Chest Hospital. A total of 52 samples were from fungal infection patients, 140 samples were from probable fungal infection patients, and 382 samples were from the fungal infection-free or healthy cohort. The samples were tested using the chemiluminescence method and the Limulus amebocyte lysate method simultaneously. The BDG testing method was performed in duplicate for each sample. The fungal (1–3)-β-D-glucan assay kit (chromogenic method) [Dynamiker (Tianjin) Biotechnology Co. Ltd] was used for the Limulus amebocyte lysate method. The chemiluminescence testing system is described in Section 2.7. ROC was calculated using SPSS, which was used for consistency evaluation for both the Limulus amebocyte lysate method and the chemiluminescence method. The sensitivity of both BDG testing method was compared (sensitivity is calculated as the ratio of positive sample amount tested by the Limulus amebocyte lysate method or the chemiluminescence method to a sample amount of fungal infection patients).

## 3 Results and discussion

### 3.1 Immunization with single-targeted antibody

**Figure 3:**
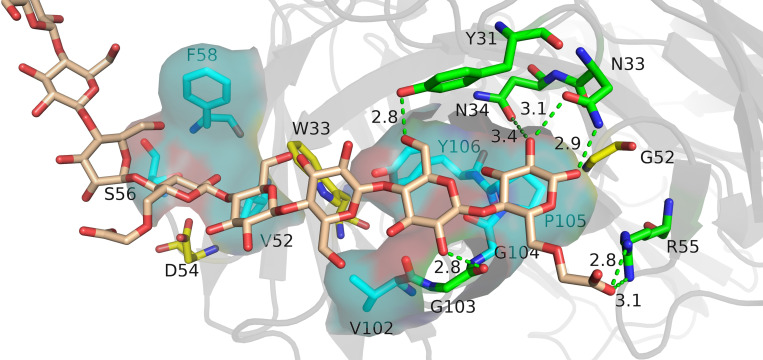
The analysis diagram of the key residues of antigen–antibody binding. Indigo represents the residues that interact with antigens through hydrophobic interaction, green represents the residues that bind to antigens through hydrogen bonds, and yellow represents residues that play an indirect role in the binding process between antigens and antibodies.

Standardized carboxymethylated pachyman (CP-BDG) was used as the immunogen to obtain WT-BDG-Ab by immunizing rabbits. Pachyman, a type of BDG from *Poria cocos*, was carboxymethylated to overcome the limitation of water solubility (Liu et al., 2021). Since the purity of the antigen will directly affect the quality of antibody, standardized antigen was prepared by semi-preparative high performance liquid chromatography (**Supplementray Figure 3**). The specificity analysis of WT-BDG-Ab (**Figure 1**) suggested that it did not produce immune responses to candida mannoprotein, aspergillus galactomannan, and cryptococcal polysaccharide, which was comparable to the background.

### 3.2 Analysis of the properties of key amino acids

**Figure 4:**
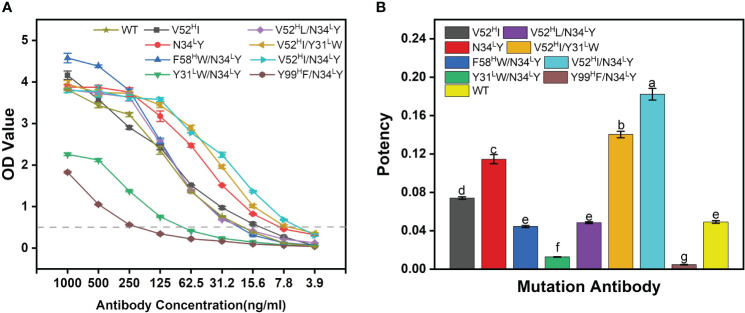
The potency comparison of BDG-Abs. **(A)** Potency graph for WT-BDG-Ab and its mutants. The antibodies were multiple proportion diluted (1,000–3.90625 ng/mL), then added to the plates coated with BDG. Then, the indirect ELISA was operated (detailed procedure is presented in Section 2.5). The potency of BDG-Abs was compared according to OD value (*n* = 3). **(B)** Comparison of the potency of WT-BDG-Ab and its mutants. The BDG-Ab potency was defined as the reciprocal of the BDG-Ab concentration at OD = 0.5. Error bars represent the standard deviations of three independent experiments. Different letters above the bars indicate statistically significant differences between the samples (one-way ANOVA followed by a *post hoc* Tukey test, *p*< 0.05).

**Figure 5:**
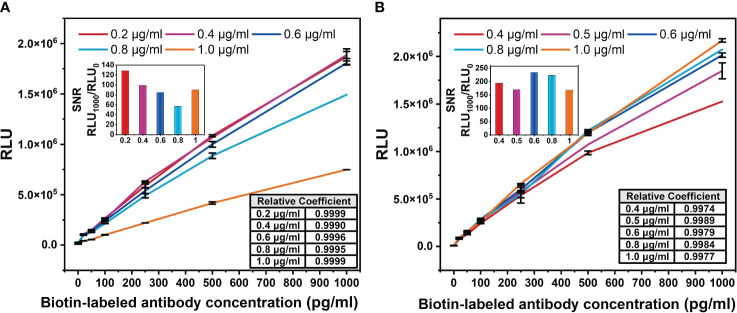
Optimization diagram of antibody concentration in chemiluminescence method. **(A)** Biotin-labeled antibody concentration. The concentration of acridine ester-labeled antibody was fixed at 0.5 μg/mL. **(B)** Acridine ester-labeled antibody concentration. The concentration of biotin-labeled antibody was fixed at 0.2 μg/mL. The signal-to-noise ratio (SNR) of the standard curve of biotin-labeled antibody with different concentration was defined as the ratio of the light value of 1,000 pg/mL antigen to the background light value. The correlation coefficients between the logarithm of BDG concentrations and light values were calculated.

Endogenous proteins are autonomously produced by the organism to meet physiological functions, which is difficult to meet the needs of clinical treatment. Therefore, studying the antigen–antibody binding mechanism is conducive to the development of BDG-Ab in clinical diagnosis. Antibody specificity is determined by the antigen binding sites in the CDR region. We obtained a large number of single-point mutant antibodies in the CDR region by alanine scanning to discuss the key sites, which are the main contributors of non-covalent bonds. They induce interactions at other sites. Therefore, mutation of key sites causes loss of non-covalent bonds through broad structural change of the interface (Fernández Quintero et al., 2020). In this study, the AI tetra-chain modeling was selected (**Supplementray Figures 4, 5**). The variable regions of one heavy chain (chain B) and one light chain (chain C) were selected for the calculated alanine scan. The general trend of the experimental alanine scans was consistent with the calculated results.

**Figure 6:**
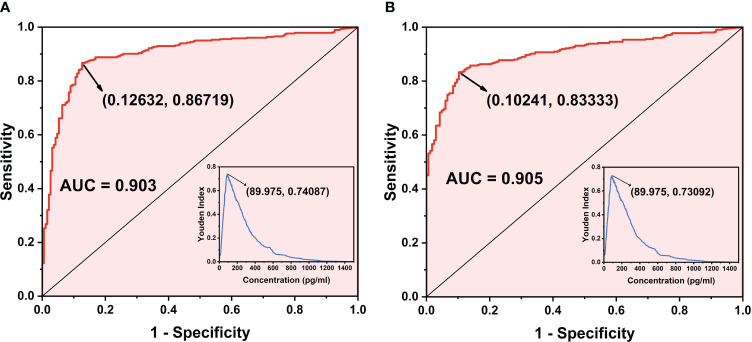
ROC curve of the chemiluminescence method. **(A)** ROC curve when the cutoff value of the Limulus amebocyte lysate detection method is 70 pg/mL as the standard. **(B)** ROC curve when the cutoff value of the Limulus amebocyte lysate detection method is 95 pg/mL as the standard.

The relative binding and mutation energy results reveal that Trp33^H^, Val52^H^, Asp54^H^, Phe58^H^, Tyr99^H^, Pro105^H^, and Tyr106^H^ residues in chain B and Tyr31^L^, Asn33^L^, Asn34^L^, Gly52^L^, and Arg55^L^ residues (the superscripts ^H^ and ^L^ indicate the heavy chain and the light chain of BDG-Ab, respectively) in chain C are the key sites in the antigen–antibody binding (**Figures 2A, B**). Antigen recognition is primarily governed by several types of non-covalent bond interactions: electrostatic interactions, van der Waals forces, hydrogen bonds, and hydrophobic interactions, while the latter is dominant (Yoshida et al., 2019). The Y106^H^A mutant had a high mutation energy of 1.22 kcal/mol and an RBC value of −29.5%, indicating that the Tyr106 residue is a “hot spot” residue in the interaction. Similarly, the mutant binding affinity of V52^H^A, F58^H^A, and P105^H^A mutants was significantly reduced (RBC values were −98.4%, −69.3%, and −29.9%, respectively). The “hot spot” residues Val52^H^, Phe58^H^, Pro105^H^, and Tyr106^H^ mostly play the hydrophobic function in the analysis of the docking data. In light chain, Y31^L^A and N34^L^A mutants had a significant decrease in binding affinity (RBC values were −101.8% and −92.7%, respectively) and had a mutation energy of 0.61 and 0.34 kcal/mol, respectively. The four “hot spot” residues Tyr31^L^, Asn33^L^, Asn34^L^, and Arg55^L^ bind the antigen mainly through hydrogen bonds, and the mutation energy also reveals that the hydrogen bonding force is weaker than the hydrophobic interaction. As shown in **Supplementray Figure 6**, Asn34^L^ has a hydrogen bond connection length of 3.4 Å, which is close to the hydrogen bond limit connection length. Moreover, the Trp33^H^, Asp54^H^, and Tyr94^L^ residues did not interact directly with the antigen, but their mutants also had a significant decrease in binding affinity. The positions of these residues were mainly distributed near “hot spot” residues (**Figure 3**), indicating that these mutants generate new interactions at the periphery of the interface to affect the antibody affinity. Although experimental and computational results for the Y99^H^A mutant show the same trend, the effects imposed by the mutation vary widely. Experiments found that the Tyr99^H^ residue interacts directly with the antigen and that mutation results in loss of antibody function, but the model of the antibody protein at this position deviated from the actual structure and produced inconsistent results.

### 3.3 Rational design of BDG-Ab

The antigen–antibody binding was dependent on the role played by the side chains of residues in the interaction (Akiba et al., 2019). Amino acid residues of the same type have similar functions. Therefore, we calculated saturation mutations for the “hot spot” residues with the same type of amino acids to identify better combinations. At most, two residues were predicted to mutate simultaneously. More mutation sites would affect the antibody’s tertiary structure more significantly, which would bias the simulation results. Meanwhile, the mutation energy of the double point mutant antibody was calculated by pairwise combination. The mutation energies of 944 combinations were calculated by mutating the 11 “hot spot” residues to the same type residues (**Supplementray Figure 7**). Most mutations lead to increased energy, exacerbating the instability of antigen–antibody binding. Therefore, we selected the eight mutation combinations with the lowest energy for antibody expression, purification, and potency testing from 16 results that had mutation energy below −0.5 kcal/mol.

Most of the mutants outperformed the WT-BDG-Ab at different antibody concentrations (**Figure 4A**). However, the detection ability varied widely at higher antibody concentrations. We used the antibody concentration that corresponded to an absorbance of 0.5 as the measure of antibody potency in this work (**Figure 4B**). The combined mutation of F58^H^W/N34^L^Y and V52^H^L/N34^L^Y was comparable in potency to the WT-BDG-Ab, while the V52^H^I, N34^L^Y, V52^H^I/Y31^L^W, and V52^H^I/N34^L^Y mutants were superior to the WT-BDG-Ab. However, antibodies with the Tyr99^H^ mutation had low binding capacity to the antigen, consistent with the experimental alanine scan results. The calculation failed to predict this mutation accurately. Secondly, the Y31^L^W/N34^L^Y double mutation also reduced the antigen–antibody affinity substantially. The Tyr31^L^ and Asn34^L^ residues are spatially close, and the mutation may have altered the conformation of the light chain domain. The experimental results showed that the V52^H^I/N34^L^Y mutant had the highest potency, which was 3.7-fold higher than that of WT-BDG-Ab.

### 3.4 Recognition ability of wild-type and mutant BDG-Ab to different fungi and bacteria

Wild-type BDG-Ab and mutant BDG-Ab display different recognition ability to different fungi and bacteria. As shown in **Tables 1** and **2**, apart from *C. neoformans*, all the testing fungi could be recognized by wild-type BDG-Ab; meanwhile, 52^H^I, N34^L^Y, F58^H^W/N34^L^Y, V52^H^I/Y31^L^W, and V52^H^I/N34^L^Y mutant BDG-Ab could recognize all the fungi being tested. Other mutants have weaker recognizing abilities to the tested fungi. All the BDG-Abs could not recognize Gram-negative bacteria, whereas wild-type, 52^H^I, N34^L^Y, F58^H^W/N34^L^Y, and V52^H^I/Y31^L^W mutant BDG-Ab could recognize *Leuconostoc mesenteroides* (a kind of Gram-negative bacterium). This may be due to the fact that *L. mesenteroides* could also produce BDG. The bacterial BDG could not be recognized by V52^H^I/N34^L^Y mutant BDG-Ab, indicating its increase in specificity of BDG-Ab. N34^L^Y single-point mutation could increase Ab potency as well. However, when combined with the Y31^L^W or Y99^H^F mutant, the antigen–antibody binding ability of Abs could be decreased significantly. The thickness of capsule of *C. neoformans* may lead to the incomplete release of BDG; thus, *C. neoformans* could be poorly recognized by BDG-Abs. Gram-negative bacterium could not be significantly recognized either by wild-type BDG-Ab or by mutant BDG-Abs, which makes a significant contribution to improve the testing specificity of BDG. The result has settled the main shortcoming of the G test, which broadens the horizon of BDG detection. Recognition of *L. mesenteroides* by wild type and some of the mutant BDG-Ab may be due to BDG production by *L. mesenteroides.* Nevertheless, V52^H^I/N34^L^Y mutant BDG-Ab could not recognize *L. mesenteroides*; the reason for this has to be further discussed.

**Table 1:** Recognition ability of wild type and mutant BDG-Ab to different fungi.

Fungus	Wild type and mutant BDG-Abs								
	Wild type	52^H^I	N34^L^Y	F58^H^W/N34^L^Y	Y31^L^W/N34^L^Y	V52^H^L/N34^L^Y	V52^H^I/Y31^L^W	V52^H^I/N34^L^Y	Y99^H^F/N34^L^Y
*Aspergillus fumigatus*	**√**	**√**	**√**	**√**	**√**	**√**	**√**	**√**	**×**
*Aspergillus niger*	**√**	**√**	**√**	**√**	**√**	**√**	**√**	**√**	**×**
*Aspergillus terreus*	**√**	**√**	**√**	**√**	**√**	**√**	**√**	**√**	**×**
*Penicillium digitatum*	**√**	**√**	**√**	**√**	**√**	**√**	**√**	**√**	**×**
*Candida albicans*	**√**	**√**	**√**	**√**	**√**	**√**	**√**	**√**	**√**
*Pneumocystis jiroveci*	**√**	**√**	**√**	**√**	**×**	**√**	**√**	**√**	**×**
*Histoplasma capsulatum*	**√**	**√**	**√**	**×**	**×**	**√**	**√**	**√**	**×**
*Cyanobacteria marneffei*	**√**	**√**	**√**	**√**	**×**	**√**	**√**	**√**	**×**
*Cryptococcus neoformans*	**×**	**√**	**√**	**×**	**×**	**×**	**√**	**√**	**×**
*Saccharomyces cerevisiae*	**√**	**√**	**√**	**√**	**×**	**√**	**√**	**√**	**×**
*Trichophyton interdigitalis*	**√**	**√**	**√**	**√**	**×**	**×**	**√**	**√**	**×**
*Cladosporium cladosporioides*	**√**	**√**	**√**	**√**	**×**	**√**	**√**	**√**	**×**

**Table 2:** Recognition ability of wild type and mutant BDG-Ab to different bacteria.

Bacteria	Wild type and mutant BDG-Abs								
	Wild type	52^H^I	N34^L^Y	F58^H^W/N34^L^Y	Y31^L^W/N34^L^Y	V52^H^L/N34^L^Y	V52^H^I/Y31^L^W	V52^H^I/N34^L^Y	Y99^H^F/N34^L^Y
*Bacillus subtilis*(G+)	**×**	**×**	**×**	**×**	**×**	**×**	**×**	**×**	**×**
*Leuconostoc mesenteroides*(G+)	**√**	**√**	**√**	**√**	**×**	**√**	**√**	**×**	**×**
*Staphylococcus aureus*(G+)	**×**	**×**	**×**	**×**	**×**	**×**	**×**	**×**	**×**
*Listeria monocytogenes*(G+)	**×**	**×**	**×**	**×**	**×**	**×**	**×**	**×**	**×**
*Escherichia coli*(G−)	**×**	**×**	**×**	**×**	**×**	**×**	**×**	**×**	**×**
*Legionella pneumophilia*(G−)	**×**	**×**	**×**	**×**	**×**	**×**	**×**	**×**	**×**
*Klebsiella pneumoniae*(G−)	**×**	**×**	**×**	**×**	**×**	**×**	**×**	**×**	**×**
*Salmonella typhimurium*(G−)	**×**	**×**	**×**	**×**	**×**	**×**	**×**	**×**	**×**
*Pseudomonas aeruginosa*(G−)	**×**	**×**	**×**	**×**	**×**	**×**	**×**	**×**	**×**
*Haemophilus influenzae*(G−)	**×**	**×**	**×**	**×**	**×**	**×**	**×**	**×**	**×**
*Haemophilus parainfluenzae*(G−)	**×**	**×**	**×**	**×**	**×**	**×**	**×**	**×**	**×**

√ represents strong recognition, **×** represents weak or no recognition, G+ represents Gram-positive bacterium, and G− represents Gram-negative bacterium.

### 3.5 Analytical capability comparison

The BDG testing ability of the chemiluminescence method was further evaluated, and the Limulus amebocyte lysate method was used as the control. The result is shown in **Table 3**. The LoB and LoD of the Limulus amebocyte lysate method are 15 pg/mL and 5 pg/mL, respectively, while the LoB and LoD of the chemiluminescence method were decreased to 5 pg/mL and 10 pg/mL for LoB and LoD, respectively. This may be due to the fact that the chemiluminescence method is an endotoxin contamination-free BDG testing method. Moreover, the chemiluminescence method could widen the linear range from 37.5–600 pg/mL to 200–1,000 pg/mL. Both two BDG testing methods share similar precision and stability.

**Table 3:** Analytical capability comparison data of the Limulus amebocyte lysate method and the chemiluminescence method.

Analytical capability	Reference	Limulus amebocyte lysate method	Chemiluminescence method
LoB	EP17 A2	15 pg/mL	5 pg/mL
LoD	EP17 A2	30 pg/mL	10 pg/mL
Precision	EP05 A3	CV of repeatability is ≤8%, CV of reproducibility is ≤12%	CV of repeatability is ≤5%, CV of reproducibility is ≤10%
Linear range	EP06 A	37.5–600 pg/mL	20–1,000 pg/mL
Stability	EP25 A	Storage at 2–8°C for 12 months	Storage at 2–8°C for 12 months

### 3.6 Clinical application of the V52HI/N34LY mutant

The V52^H^I/N34^L^Y mutant was applied to a chemiluminescent platform. Chemiluminescence assay does not require immobilizing the primary antibody on the ELISA plate, unlike the ELISA double-antibody sandwich assay. This enables more complete interaction between the free antibodies and the antigen, resulting in better detection. Both the biotin-labeled and acridine ester-labeled antibodies target the same antigen. Firstly, the optimal concentration of the biotin-labeled antibody was determined. The concentration of the acridine ester-labeled antibody was fixed at 0.5 μg/mL, then different concentrations of the biotin-labeled antibody were used to detect the gradient concentration of antigen, and the standard curve was plotted. **Figure 5A** has shown that the increase of the biotin-labeled antibody occupied a large number of the epitopes on BDG, and the light value of 1 μg/mL biotin antibody decreased by about half at the same BDG concentration. The maximum light value gradually entered the plateau period as the biotin-labeled antibody addition decreased, and basically ceased to change at 0.4 μg/mL.

In order to accurately detect the actual concentration of antigen and increase the threshold of detectable light value, the signal-to-noise ratio (SNR) of the standard curve of the biotin-labeled antibody with different concentrations was calculated. We defined the SNR as the ratio of the light value of 1,000 pg/mL antigen to the background light value. Among the five concentrations, the biotin-labeled antibody at 0.2 μg/mL had the highest SNR (**Figure 5A**), which was used for the subsequent study. The light value increased with the concentration of the acridine ester-labeled antibody (**Figure 5B**), and we selected the concentration of 0.6 μg/mL with the highest SNR for the subsequent study. The correlation coefficients between the logarithm of BDG concentrations and the light values were greater than 0.99, indicating a strong correlation.

A total of 574 clinical samples were tested using the developed method and the Limulus amebocyte lysate method, respectively. The Limulus amebocyte lysate method can accurately identify patients with BDG concentrations above 95 pg/mL as positive and below 70 pg/mL as negative, but not those with BDG concentrations between 70 and 95 pg/mL. The ROC curves of the chemiluminescence method (**Figures 6A, B**) were plotted using the Limulus amebocyte lysate method as the cutoff values for negativity and positivity. With a cutoff value of 70 pg/mL, the chemiluminescence method had an area under the curve (AUC) of 0.903 (95% CI: 0.876–0.930) and a maximum Youden index of 0.7409. The antigen concentration detected by the chemiluminescence method was 89.98 pg/mL, which was within the 70–95 pg/mL range. The sensitivity and specificity of the chemiluminescence method were 86.72% and 87.37%, respectively, using this antigen concentration as the cutoff value. With a cutoff value of 95 pg/mL, the AUC of the chemiluminescence method was 0.905 (95% CI: 0.880–0.929), which was higher than the cutoff value of 70 pg/mL, with a maximum Youden index of 0.7309, and the antigen concentration was 89.98 pg/mL. The sensitivity and specificity were 83.33% and 89.76%, respectively, with the antigen concentration cutoff value of 89.98 pg/mL. In addition, compared with the Limulus amebocyte lysate method, the chemiluminescence method has lower sensitivity (**Table 4**).

**Table 4:** Sensitivity comparison between the chemiluminescence method and the Limulus amebocyte lysate method.

Sample type	Sample amount	Result	Testing method	
			Limulus amebocyte lysate method	Chemiluminescence method
			Amount	Amount
Serum from fungal infection patients	52	Positive	46	46
		Sensitivity	88.5%	88.5%
Serum from probable fungal infection patients	140	Positive	116	120
		Sensitivity	82.9%	85.7%

The existing technologies for the quantitation of BDG is limited. The Limulus amebocyte lysate method relies on cells derived from the blood of horseshoe crabs, which may lead to the population decline of horseshoe crabs. Compared to the Limulus amebocyte lysate method, chemiluminescence is free of endotoxin contamination. Last but not least, such method has a sustainable raw material source, which offers the testing method a long-term development.

In this study, a V52^H^I/N34^L^Y mutant BDG-Ab was discovered for the construction of a chemiluminescence-based BDG testing system. According to the clinical sample testing result, the chemiluminescence-based method possesses a similar clinical compliance rate with the Limulus amebocyte lysate method and better sensitivity and specificity. The operation protocol is practically simple since the chemiluminescence-based method is a direct “mix-to-test” method without a washing procedure. Based on such reasons, the chemiluminescence-based method is a better choice for BDG testing.

## 4 Conclusion

In the study, a high-performance mutant antibody was generated by immunizing rabbits with purified CP-BDG and experimental as well as computational alanine scanning on the CDR region of BDG-Ab. The V52^H^I/N34^L^Y mutant exhibited a 3.7-fold higher potency than WT-BDG-Ab, which was labeled with biotin and acridine ester for the chemiluminescence assay. Then, a novel detection method for BDG based on a magnetic particle chemiluminescence platform using V52HI/N34LY mutant was developed. Compared to the Limulus amebocyte lysate method, the chemiluminescence method showed a lower LoB and LoD, a wider linear range, and better sensitivity, which poses accurate quantitation for clinical sample testing. The assay demonstrated high sensitivity and specificity (83.33% and 89.76%, respectively) for detecting BDG in serum, with an antigen concentration cutoff value of 89.98 pg/mL. The results indicated that the developed method could serve as an alternative to the Limulus amebocyte lysate assay and enable a rapid and accurate diagnosis of IFD.

## Data availability statement

The original contributions presented in the study are included in the article/**Supplementary Material.** Further inquiries can be directed to the corresponding authors.

## Ethics statement

The study was approved by the Medical Ethics Committee of Shandong Chest Hospital under the registration number 2020XKYYEC-48. The animal study was approved by Animal Ethics Committee of Tianjin Fifth Central Hospital under the registration number TJWZX2018048. The study was conducted in accordance with the local legislation and institutional requirements.

## Author contributions

CL: Conceptualization, Methodology, Validation, Writing – original draft. LZ: Investigation, Methodology, Visualization, Writing – review & editing. JZ: Conceptualization, Methodology, Software, Writing – review & editing. MW: Formal analysis, Methodology, Writing – review & editing. SY: Conceptualization, Writing – review & editing. RS: Resources, Supervision, Writing – review & editing. WQ: Project administration, Writing – review & editing.
