# Supplementary material for: Rational design of antibodies and development of a novel method for (1–3)-β-D glucan detection as an alternative to Limulus amebocyte lysate assay
Source: Front Cell Infect Microbiol. 2024 Jan 24;14:1322264. doi: 10.3389/fcimb.2024.1322264 (PMC10847287; doi:10.3389/fcimb.2024.1322264)
Supplement: Supplementary file 1 [file DataSheet_1.docx]

Supplementary Material

**Table S1.** Amino acid sequence of BDG-Ab

| Name | N-C |
| --- | --- |
| BDG-Ab-H | QSLQESGGGLVQPGASLTLTCKASEFSFNNDFWICWVRQAPGKGLEWIACMVPDGSGFGFSASWAKGRFTISRTSSTTMTLQMTSLTAADTATYFCTRYGDVGGPYSFKIWGPGTLVTVSS  GQPKAPSVFPLAPCCGDTPSSTVTLGCLVKGYLPEPVTVTWNSGTLTNGVRTFPSVRQSSGLYSLSSVVSVTSSSQPVTCNVAHPATNTKVDKTVAPSTCSKPTCPPPELLGGPSVFIFPPKPKDTLMISRTPEVTCVVVDVSQDDPEVQFTWYINNEQVRTARPPLREQQFNSTIRVVSTLPIAHQDWLRGKEFKCKVHNKALPAPIEKTISKARGQPLEPKVYTMGPPREELSSRSVSLTCMINGFYPSDISVEWEKNGKAEDNYKTTPAVLDSDGSYFLYSKLSVPTSEWQRGDVFTCSVMHEALHNHYTQKSISRSPGK |
| BDG-Ab-L | AAVLTQTPSPVSAAVGGTVTISCQSSQSVGYGNNLAWYQQKPGQPPKLLIYGASRLASGVPSRFSGSGSGTQFTLTINGVQCDDAATYYCAGDYGIITDMCVFGGGTEVVVK  GDPVAPTVLIFPPAADQVATGTVTIVCVANKYFPDVTVTWEVDGTTQTTGIENSKTPQNSADCTYNLSSTLTLTSTQYNSHKEYTCKVTQGTTSVVQSFNRGDC |

**Table S2.** Amino acid sequence of CDR and FR regions

| Name | N-C |
| --- | --- |
| FR1-H | QSLQESGGGLVQPGASLTLTCKASEFSFN |
| FR1-L | AAVLTQTPSPVSAAVGGTVTISC |
| CDR1-H | NDFWIC |
| CDR1-L | QSSQSVGYGNNL |
| FR2-H | WVRQAPGKGLEWIA |
| FR2-L | WYQQKPGQPPKLLIY |
| CDR2-H | CMVPDGSGFGFSASWAKG |
| CDR2-L | GASRLAS |
| FR3-H | RFTISRTSSTTMTLQMTSLTAADTATYFCTR |
| FR3-L | GVPSRFSGSGSGTQFTLTINGVQCDDAATYYC |
| CDR3-H | YGDVGGPYSFKI |
| CDR3-L | AGDYGIITDMCVF |
| FR4-H | WGPGTLVTVSS |
| FR4-L | GGGTEVVVK |

**
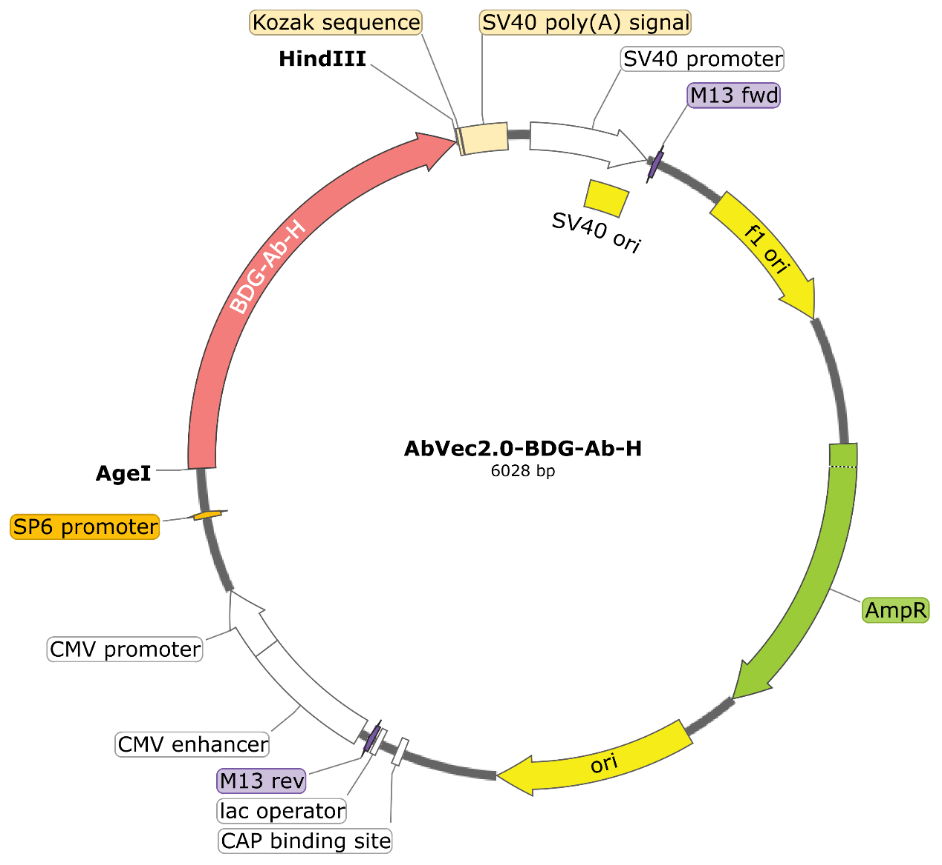
**

**Figure S1.** The Plasmid map of AbVec2.0-BDG-Ab-H. Constructs carrying the BDG-Ab-H synthesis pathway genes on the AbVec2.0 plasmid by inserting BDG-Ab-H whole segment genes into the AgeI/HindIII sites. Each segment of the gene was initiated by a SP6 promoter.

**
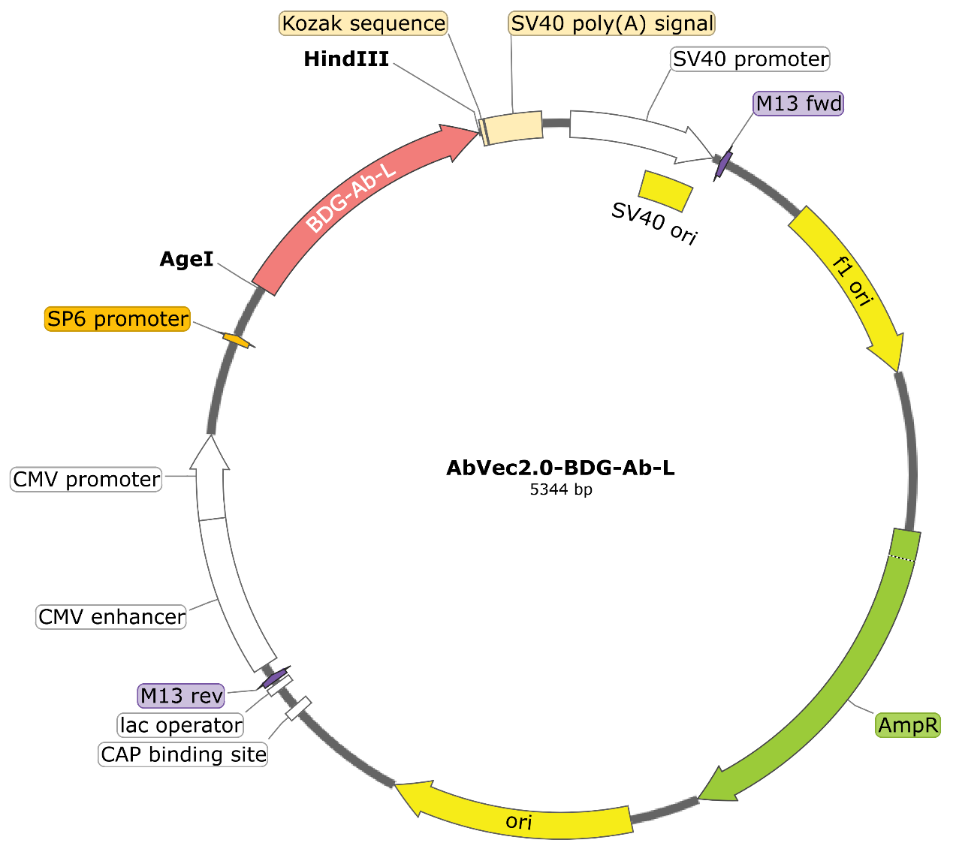
**

**Figure S2.** The Plasmid map of AbVec2.0-BDG-Ab-L. Constructs carrying the BDG-Ab-L synthesis pathway genes on the AbVec2.0 plasmid by inserting BDG-Ab-L whole segment genes into the AgeI/HindIII sites. Each segment of the gene was initiated by a SP6 promoter.


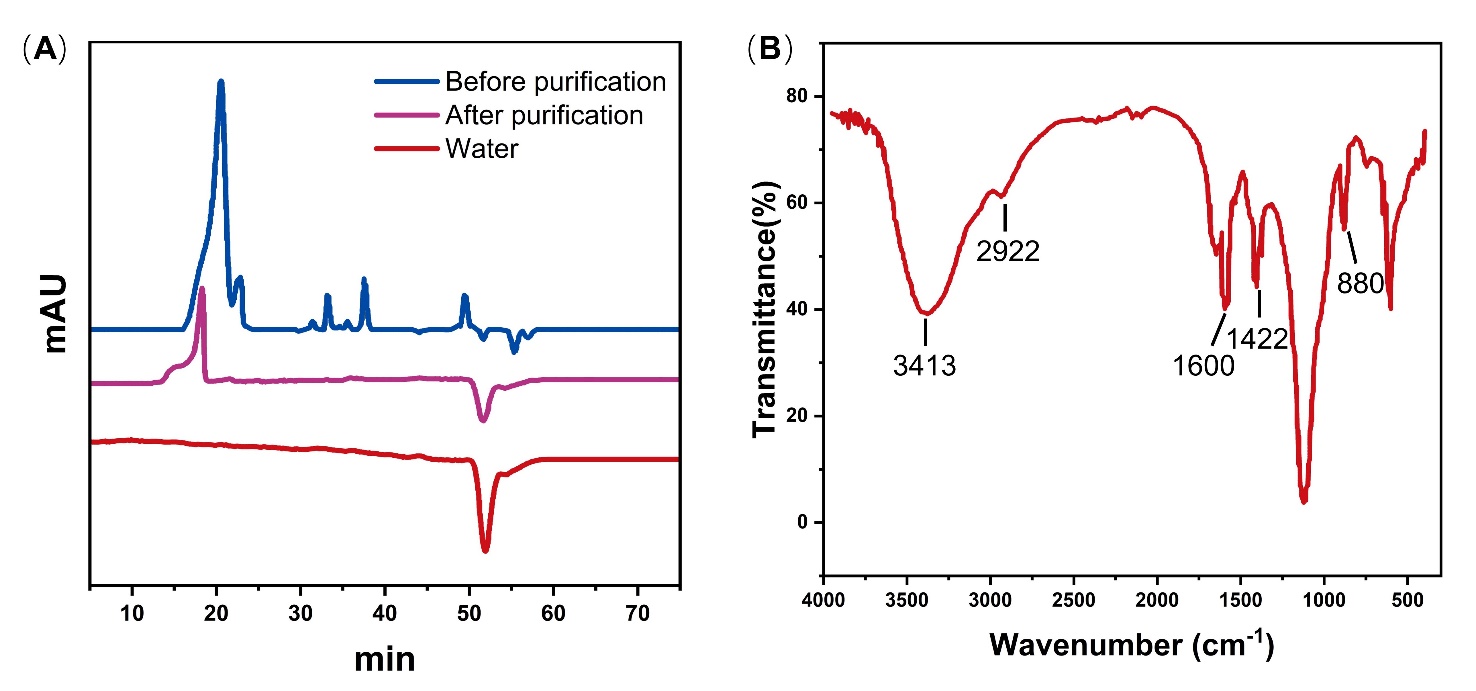


**Figure S3.** Characterization diagram of Carboxymethylated pachyman (CP-BDG). (**A**) High performance liquid chromatography analysis chart. (**B**) Fourier transform infrared spectrogram.

CP-BDG was purchased from Megazyme. The CP-BDG sample was dissolved in endotoxin-free water, and then boil for 3 hours. The suspension was cooled to room temperature, centrifuged at 3000 rpm for 10 min, and the supernatant was collected to TSKgel G2500PWxl (7.8 mm I.D. × 300 mm, 7 μm), Agilent 1200 liquid chromatography with differential refractive index detector detection. The main peak that appears around 20 min was collected. Fourier transform infrared (FTIR) analysis was carried out by Nicolet 6700 Fourier transform Infrared Spectrometer (Nicolet Company, USA).

The peaks at around 341 and 2922 cm^-1^ can be assigned to the *v*(O-H) and *v*(C-H) stretching vibrations respectively(1), which were typical characteristic absorption peaks of glycans. The peak at around 1095 cm^-1^ was the stretching vibration of pyranose rings in the β glucosyl residue(2, 3), and the peak at around 880 cm^-1^ was characteristic of β-terminal isomeric *δ*(C-H) vibration absorption peak(4), which indicated the presence of β-glycosidic linkage. The peaks at around 1600 and 1422 cm^-1^ can be assigned to the *v*_as_(C=O) asymmetric and *v*_s_(C=O) symmetrical stretching vibrations respectively(5), which were the characteristic absorption peak of carboxymethyl bond.


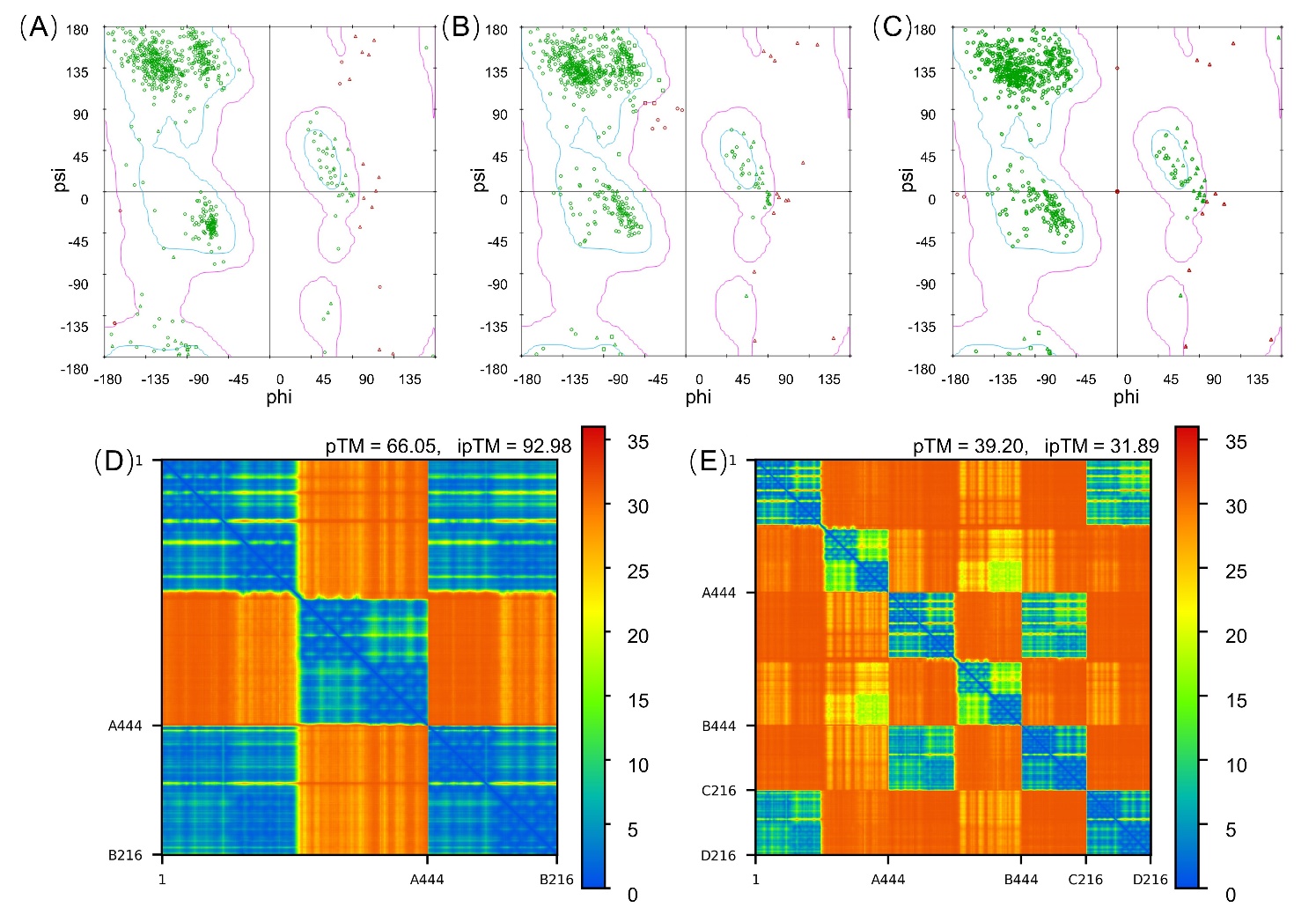


**Figure S4.** Evaluation diagram of the three-dimensional structural model of BDG-Ab. (**A**) Ramachandran plot of homologous modeling BDG-Ab, 5 amino acids except proline are in the disallowable region. (**B**) Ramachandran plot of AI double-chain modeling BDG-Ab. (**C**) Ramachandran plot of AI four-chain modeling BDG-Ab. The amino acids in the maximum allowable region in (B) and (C) had significantly more quantity and more concentrated positions, the maximum number of amino acids in the maximum allowable region was reached in (C). (**D**) The predicted aligned error (PAE) plot of AI double-chain modeling BDG-Ab. The heavy chain and the light chain correspond to chain A and B, respectively. (**E**) PAE plot of AI four-chain modeling BDG-Ab. Two heavy chains correspond to chain A and B and two light chains correspond to chain C and D, while the two variable regions of BDG-Ab consist of chain A, D and chain B, C, respectively. For the light chains and half of heavy chains, the PAE is small and the relative orientation is predicted to be accurate. In contrast, the other parts of the heavy chains have a large PAE relative to the other domains because this domain structure is not resolved in the same crystal.


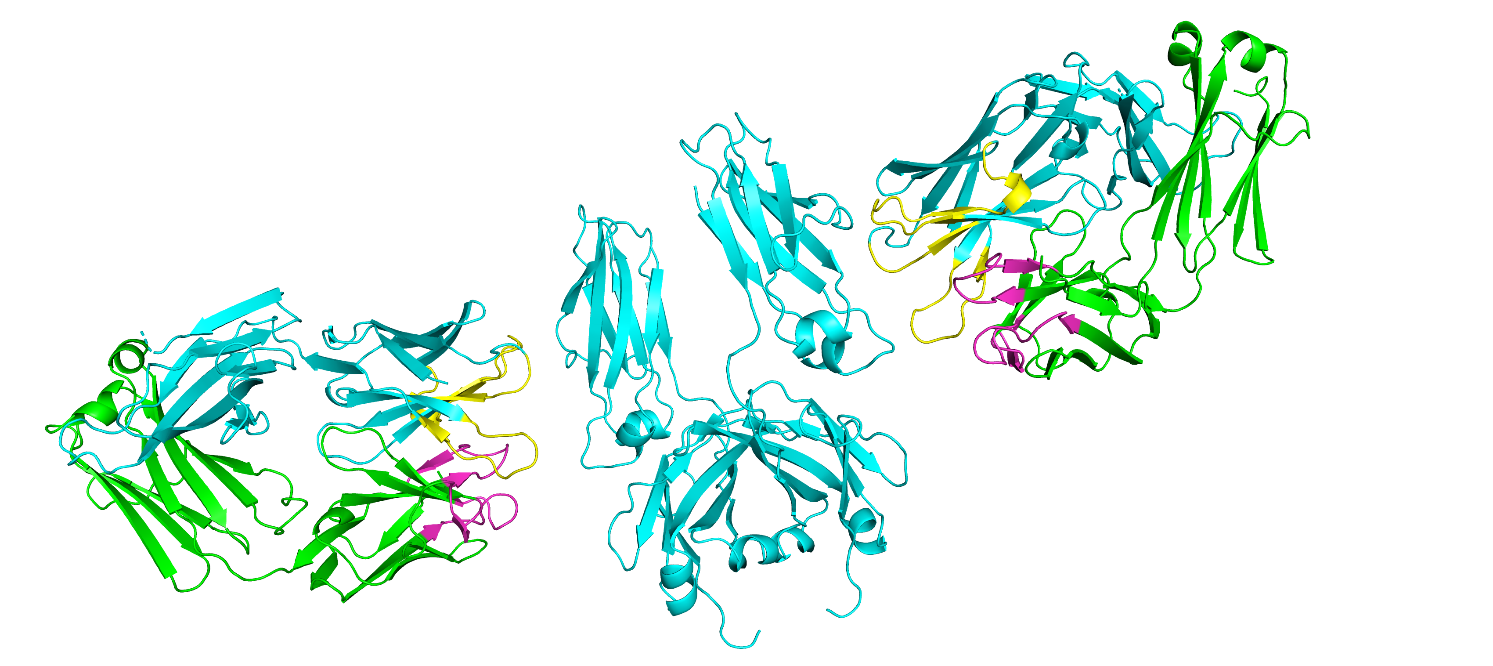


**Figure S5.** Three-dimensional structural model of BDG-Ab with AI tetra-chain modeling. Indigo represents the BDG-Ab-H, yellow represents the CDR regions on heavy chain, green represents the BDG-Ab-L, and carnation represents the CDR regions on light chain.


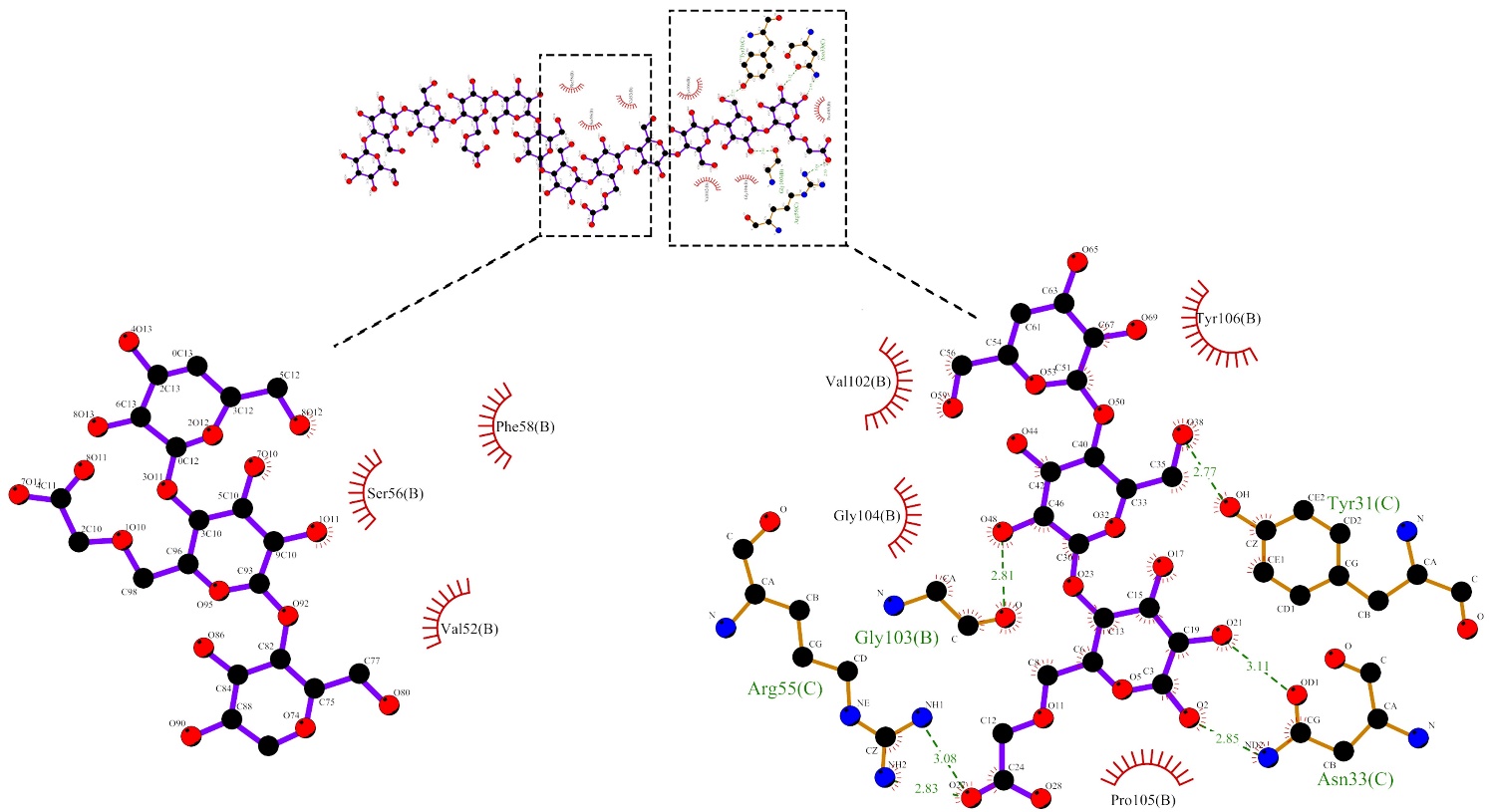


**Figure S6.** Two-dimensional diagram of antigen and antibody binding.


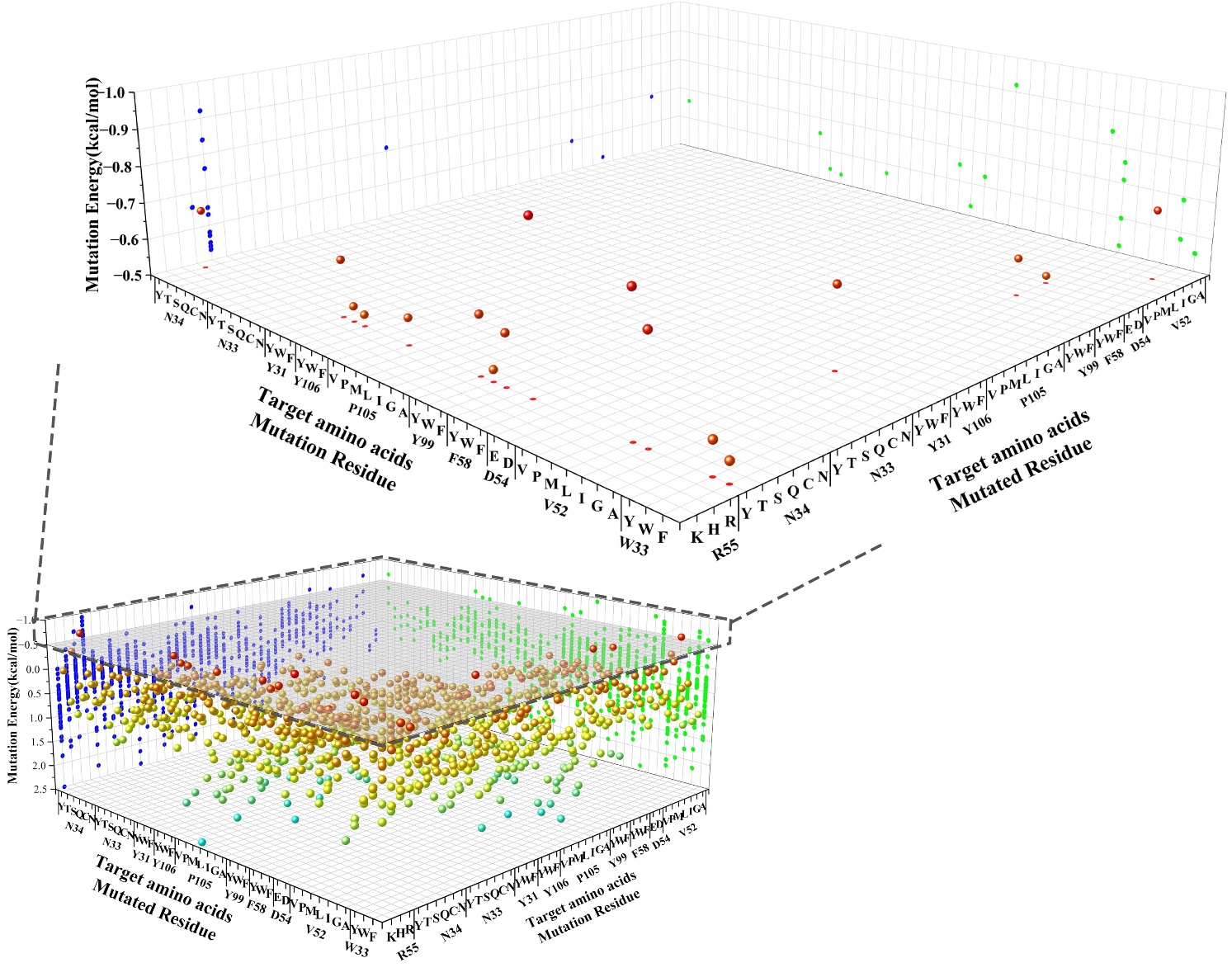


**Figure S7.**  Energy diagram of saturation mutations of key residues with the same type of amino acid. Red ball represents the mutation energy of mutant less than -0.5 kcal/mol.

**References**

1. Liu F, Liu Y, Feng X, Ibrahim SA, Huang W. Structure characterization and in vitro immunomodulatory activities of carboxymethyl pachymaran. INT J BIOL MACROMOL. 2021;178:94-103.

2. Wu M, Feng H, Song J, Chen L, Xu Z, Xia W, et al. Structural elucidation and immunomodulatory activity of a neutral polysaccharide from the Kushui Rose (Rosa setate x Rosa rugosa) waste. CARBOHYD POLYM. 2020;232:115804.

3. Xu Z, Wang H, Wang B, Fu L, Yuan M, Liu J, et al. Characterization and antioxidant activities of polysaccharides from the leaves of Lilium lancifolium Thunb. INT J BIOL MACROMOL. 2016;92:148-55.

4. Chen L, Huang G. The antioxidant activity of derivatized cushaw polysaccharides. INT J BIOL MACROMOL. 2019;128:1-4.

5. Šandula J, Kogan G, Kačuráková M, Machová E. Microbial (1→3)-β-d-glucans, their preparation, physico-chemical characterization and immunomodulatory activity. CARBOHYD POLYM. 1999;38(3):247-53.
